# The Arizona Prevention Research Center partnerships in Arizona to promote COVID-19 vaccine health equity

**DOI:** 10.3389/fpubh.2022.944887

**Published:** 2022-07-25

**Authors:** Tomas Nuño, Lidia Azurdia Sierra, Ada Wilkinson-Lee, Scott Carvajal, Jill de Zapien, Kiera Coulter, Carlos Figueroa, Mario Morales, Ramses Sepulveda, Refugio Sepulveda, Maia Ingram

**Affiliations:** ^1^Department of Epidemiology and Biostatistics, Mel and Enid Zuckerman College of Public Health, University of Arizona, Tucson, AZ, United States; ^2^Division of Public Health Practice and Translational Research, Mel and Enid Zuckerman College of Public Health, University of Arizona, Phoenix, AZ, United States; ^3^Department of Health Promotion Sciences, University of Arizona Mel and Enid Zuckerman College of Public Health, Tucson, AZ, United States; ^4^Department of Mexican American Studies, College of Social and Behavioral Sciences, University of Arizona, Tucson, AZ, United States

**Keywords:** COVID-19, vaccine, health equity, Latinx, partnerships, collaborations

## Abstract

**Background:**

Vaccine hesitancy in the face of the COVID-19 pandemic is a complex issue that undermines our national ability to reduce the burden of the disease and control the pandemic. The COVID-19 pandemic revealed widening health disparities and disproportionate adverse health outcomes in terms of transmission, hospitalizations, morbidity and mortality among Arizona's Latinx rural, underserved, farmworker, disabled and elderly populations. In March 2021, ~8.1% of those vaccinated were Latinx, though Latinxs make up 32% of Arizona's population. The Arizona Vaccine Confidence Network (AzVCN) proposed to leverage the expertise of the Arizona Prevention Research Center (AzPRC) and the resources of the Mel and Enid Zuckerman College of Public Health (MEZCOPH) Mobile Health Unit (MHU) to identify, implement and evaluate a MHU intervention to increase uptake of COVID-19 vaccines.

**Methods:**

The AzVCN focused efforts on Latinx, rural, un/underinsured and farmworker communities in the four Arizona border counties that are at greater risk of COVID-19 morbidity and mortality and may have limited access to vaccination and other essential health services. The AzVCN used listening sessions to create a feedback loop with key stakeholders and critical health care workers to validate barriers/enablers and identify solutions to increase vaccination uptake emerging from the network. The AzVCN also implemented a community-based intervention using community health workers (CHWs) based in a MHU to increase knowledge of the COVID-19 vaccines, reduce vaccination hesitancy and increase vaccination uptake among Latinx rural, un/underinsured and farmworker populations in Southern Arizona.

**Results:**

AzVCN outcomes include: identification of enablers and barriers of COVID-19 vaccination in the priority populations; identification of strategies and solutions to address vaccine hesitancy and increase vaccine uptake among priority population; and evidence that the proposed solutions being tested through the AzVCN contribute to increased vaccine uptake among the priority populations.

**Conclusion:**

Through these efforts the AzPRC contributed to the CDC's Vaccinate with Confidence Strategy by collaborating with CHWs and other key stakeholders to engage directly with communities in identifying and addressing structural and misinformation barriers to vaccine uptake.

## Introduction

The COVID-19 pandemic revealed widening health disparities and disproportionate adverse health outcomes in terms of transmission, hospitalizations, morbidity and mortality among Arizona's rural, Hispanic/Latinx (henceforth referred as Latinx), American Indian, and elderly populations (1). Unfortunately, these were among the very populations with lower rates of COVID-19 vaccination in the early weeks of Arizona's COVID-19 vaccination rollout. In March 2021, ~8.1% of those vaccinated were Latinx and 1.1% were Native American although Latinxs make up 32% of Arizona's population and 5.3% are Native American (1). By May 2022 in Arizona, it was still below the population makeup of those groups, with ~20% of those receiving at least one COVID-19 vaccination dose being Latinx and 4.5% being Native American (2). Intractable health inequities relate to social determinants including socioeconomic status, lack of insurance, rural locations, limited English speaking skills, immigration status, unreliable transportation, difficulty obtaining childcare and other factors (3). Stigma, ageism, racism, and anti-immigrant policies further impede access to COVID-19 testing and vaccination (4). While county health departments (CHDs) responded by initiating pop-up clinics and other efforts, delays in COVID-19 vaccine availability exacerbated vaccine hesitancy in communities that were already mistrustful of health systems. In general, across the United States, there is a mistrust of vaccines and can be barriers to COVID-19 vaccinations, especially among racial/ethnic minority groups (5). In Arizona, findings from racial/ethnic minority focus groups found that COVID-19 vaccine hesitancy is multi-faceted, influenced by personal perceptions of vaccines, family and community relationships, and historical and structural factors (6). Among Latinx participants, religiosity was a key factor contributing to either vaccine hesitancy or confidence behaviors (6). Overall, lack of a unified message from the health care community, propagation of misinformation about the virus and the vaccine, long-standing distrust of vaccines, and structural barriers in the medical system all contributed to vaccine hesitancy (7).

Tailored interventions that address structural barriers for Latinx un/underinsured, farmworker and rural communities are essential to increasing COVID-19 vaccine availability and addressing vaccine hesitancy in Arizona. A key component also includes addressing negative emotions associated with the COVID-19 vaccine (8). Spanish speaking staff and providers who can communicate the importance and safety of the COVID-19 vaccines are a critical piece of the solution (9), as are trusted individuals such as community health workers (CHWs) who have an enduring presence in helping connect community members to services (10).

The Vaccine Confidence Network (VCN) is a Centers for Disease Control and Prevention (CDC) effort funded through Prevention Research Centers (PRCs) nationwide to address COVID-19 vaccine hesitancy and uptake. Originally called the Connecting Behavioral Science to COVID-19 Vaccination Demand Project (AZ CBS-CVD), this project leverages the expertise of PRCs nationwide. In Arizona, the Arizona Prevention Research Center (AzPRC) named our team the Arizona Vaccine Confidence Network (AzVCN). In partnership with the Mel and Enid Zuckerman College of Public Health (MEZCOPH) Mobile Health Unit (MHU) and the Refugees and Immigrants Community for Empowerment (RICE), project activities to address COVID-19 vaccine hesitancy were conducted to address structural and misinformation barriers that influence vaccine health equity, with a goal to increase uptake of COVID-19 vaccines among underserved Latinx communities.

## Methods

### Study team

The AzVCN was led by investigators from the AzPRC, funded by the CDC. To better understand the assets and needs of our priority communities, the AzPRC works closely with a Community Action Board (CAB). The CAB is composed of 25 organizations and programs that share a common agenda of improving the quality of life in the border region. CAB members are responsible for guiding AzPRC activities and have expressed their support and commitment to expand upon our foundation of: (i) Developing and disseminating evidence-based strategies to address disparities in health promotion and disease prevention using the CHW model and (ii) Promoting health through environmental and systems change strategies on both a local and state level. CAB members provide feedback on project design and dissemination.

The AzPRC strives to address chronic disease health disparities in underserved populations in Southern Arizona. The Southern Arizona region includes four U.S. counties that lie on the U.S.-Mexico Border: Cochise, Pima, Santa Cruz, and Yuma. The AzPRC has been working with communities along this 389-mile-long border for over 20 years. Partner communities include: Douglas, Nogales, and Somerton/San Luis, as well as the Tohono O'Odham Nation which has lands that extend from just south of Casa Grande, through western Pima County and into Mexico.

### Study design

In May 2021, the CDC's PRC network program awarded supplemental funding to all 26 PRCs in the United States to support the implementation of the CDC's COVID-19 vaccine confidence strategy. The CDC created the VCN to identify key behavioral insights to inform effective solutions to increase COVID-19 vaccine confidence and ultimately uptake. The VCN initiated as a thematic network of PRCs. The focus of the network was to more effectively translate best practices from behavioral science to improve immunization programming. The goal of the VCN was to conduct community-based evaluations to identify communities of focus, diagnose social and behavioral drivers of vaccine uptake, and design, implement, and scale up effective interventions to increase vaccine confidence and uptake at multiple levels. The broad geographic reach, diverse target populations, and strong relationships among VCN investigators at each PRC allowed the network to achieve a larger impact than any other individual PRC could achieve on its own. The guiding principle was that promoting confidence in vaccines requires more than messages. It requires commitments to listening, understanding, collaborating within communities, and changing how health services are delivered to better address the needs of individuals and communities. The study was determined to be “Exempt” by the University of Arizona Human Subjects Protection Program.

### Priority population

**Figure 1:**
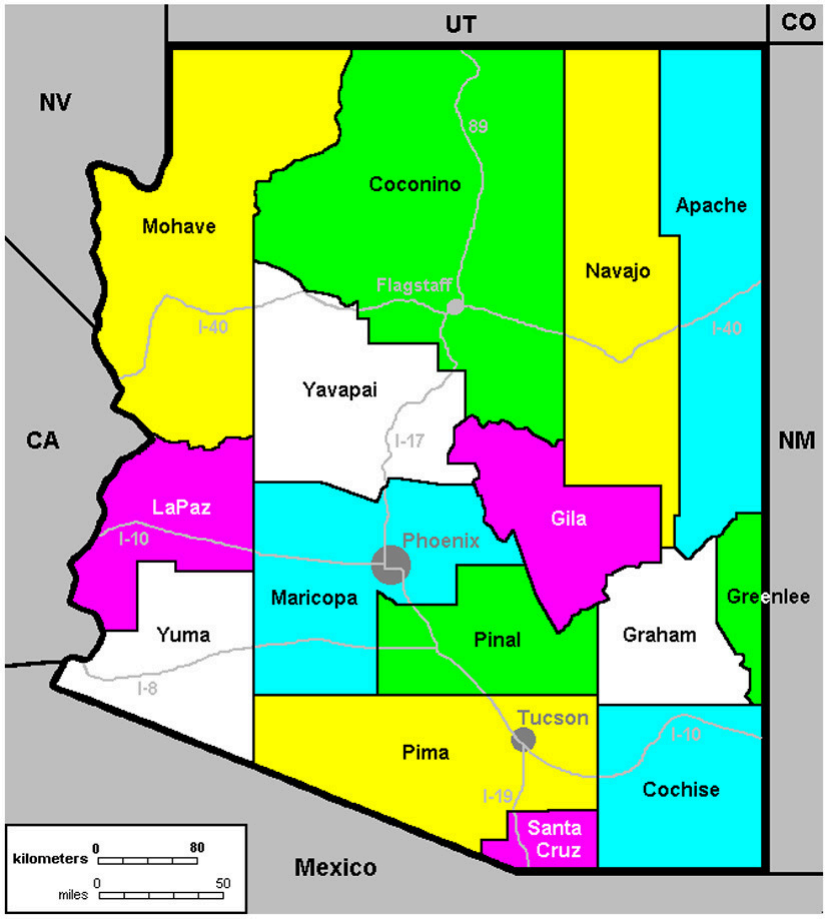
Arizona counties.

The AzVCN targeted rural, un/underinsured and farmworker communities in the four counties that make up the Arizona-Mexico border region (Figure 1). These counties have significant Latinx populations (30–80%), mostly of Mexican origin, that experience underlying social and economic disparities that create higher risk of contracting COVID-19, as well as complications due to existing conditions such as hypertension (11, 12). Border residents are twice as likely to live in poverty, be uninsured, and experience higher rates of unemployment than the population of any individual U.S. state (13). These social determinants translate directly into social and economic contexts that create barriers to accessing health care, including vaccinations, beyond cost and lack of insurance. Farmworkers and other essential workers may face financial hardship from taking a day off work to get vaccinated or worry about losing work due to the ill effects of the vaccine response. A history of poor interpersonal interactions with health providers may exacerbate reluctance to seek the COVID-19 vaccine (14).

### Data collection

Project data was collected in two primary methods. First, listening sessions with key stakeholders and critical health care workers were conducted in the Fall of 2021. The goal of the listening session was to create a feedback loop with these key stakeholders and critical health care workers that would validate barriers/enablers and identify solutions to increase COVID-19 vaccination uptake emerging from the network. The key stakeholders and critical health care workers were contacted by the study team leader *via* email to inquire about their willingness to participate in listening sessions. Second, at selected MHU community and vaccination events, CHWs provided MHU visitors with the opportunity to participate in a survey on vaccination intention/experience, enablers and barriers, and the intentions of family members. Events where the surveys were offered were selected based on availability of student interns to administer the survey and design to reach different regions of Southern Arizona. The survey consisted of tools made available through the national VCN network. The student interns administered the anonymous surveys, with data collected housed in a database separate from vaccine registration. Given that the MHU is able to provide services in diverse communities, we were able to collect data from people of all ages, in rural and urban settings, and with farmworkers and other essential workers. These data are essential in designing outreach as COVID-19 vaccines becomes more widely available.

### Outcomes and statistical analysis for the evaluation plan

The primary outcomes of interest for this project include qualitative and quantitative data. Qualitative data were collected from listening sessions with stakeholders and critical health care workers. Quantitative data will include vaccine intention and uptake variables. Variables of interest include demographics, vaccine knowledge, attitudes, and beliefs, enablers and barriers, comorbidities, and personal and family impacts. Data are stored in the HIPAA-compliant University of Arizona REDCap database program. Data will be exported to Stata 16 files (StataCorp, College Station, TX) for statistical analyses. Results of the ongoing quantitative survey will be tabulated upon conclusion of the survey data collection in August 2022.

## Results

### AzVCN activities

The AzVCN activities were designed to facilitate the identification and translation of effective strategies to implement COVID-19 immunization confidence and uptake. The activities have contributed to three overarching focus areas: (1) collecting data for action; (2) building the evidence base to increase COVID-19 vaccine confidence and uptake; and (3) evaluating solutions and increase community engagement. The AzVCN has been contributing to national PRC collaborative efforts to develop and utilize common data measures, aggregate data, and analyze data across sites, and develop best practice toolkits and social marketing materials.

### Listening sessions

Specific activities in Arizona included listening sessions to create a feedback loop with key stakeholders and critical health care workers to validate barriers/enablers and identify solutions to increase COVID-19 vaccination uptake emerging from the network. The AzVCN implemented listening sessions starting with key stakeholders from Arizona-Mexico border counties and with the AzPRC CAB that is made up of representatives from CHDs, CHW organizations, federally qualified health centers (FQHCs), and grassroots organizations. We identified other stakeholder groups including critical healthcare providers who are interfacing with the priority communities. Our relationship to CHDs was critical in prioritizing the communities engaged in this project.

The AzVCN implemented listening sessions with key stakeholders, including the AzPRC community action board (CAB) that is made up of representatives from CHDs, CHW organizations, federally qualified health centers (FQHCs), and grassroots organizations. Five listening sessions were conducted in the summer and fall of 2021. Listening sessions were conducted with two Arizona County Public Health Department Directors from two different counties, a staff member from one county, two PRC staff, four Community Health Workers, three employees of an Area Health Education Center, and fifteen CAB members. All were adults over the age of 18. Four listening sessions were conducted *via* online Zoom meetings: two county sessions, one health education center session, and one CHW session. The CAB listening session was conducted in-person at an AzPRC quarterly CAB meeting. Summaries of the listening sessions are shown in Table 1. Results of note included several references in each session to misinformation (either through social media or among peers), improved need for consistent messaging, need to focus on youth, and constantly changing information.

**Table 1:** Listening session summaries.

**Agency**	**Date**	**Participants**	**Barriers cited**	**Faciliators cited**
Yuma county public health services district	9/9/21	2	• Farmworker employment • Misinformation • Sustainablity • Phrasing of booster doses • Funding to continue to do the work	• Time off work for vaccinations • Partnerhships • Communication • Bilingual staff
Southeast Arizona health education center	9/16/21	4	• Misinformation • Sustainablity • Hesitancy among rural population	• Collaborative Partnerhships • Communication • Bilingual staff • Testimonial videos in native language
Pima county and mariposa county community health workers	9/17/21	4	• Misinformation • Need for information in Spanish • Need to reach youth • Fear of being deported • Scheduling appointments	• Use of social media targeted to youth • Education efforts by CHWs • Trust
Cochise county health department	9/22/21	2	• Motivation for vaccination falling • Social media misinformation • Anti-vaccine groups (older, rural) • Allocation of resources • Non-specific public health messaging	• Trained staff • Communication • Targeted public health messaging • Mandates
AzPRC community advisory board	12/10/21	15	• Misinformation creates hesitancy • Social media • Young people feeling invincible • Keeping messaging constant • Constantly changing information • Convincing those that personally affected by COVID-19	• Cultural facilitators • Mixed methods strategy (policy, messaging) • CHWs provding binational information • Call centers to combat misinformation • Simple messaging more effective

### Mobile health unit

The AzVCN partnered with the MEZCOPH to implement a community-based intervention using CHWs based in a MHU to address structural barriers and increase knowledge of COVID-19 vaccines, with a goal to reduce vaccination hesitancy and increase vaccination uptake among Latinx rural, un/underinsured and farmworker populations in Southern Arizona. MHUs units are effective in reducing structural, economic and social barriers to accessing health care service among our priority populations (15).

The MEZCOPH MHU conducts vaccine outreach and education in priority communities and allows for drop-in visits for COVID-19 vaccinations. In particular, the MHU partners with local county health departments to set up vaccination or health information events on a monthly basis. During the waiting periods before and after the vaccine, CHWs provide on-site education on COVID-19 transmission and prevention strategies to protect family members living in the same household. The MHU also refers residents to other health and social services. The program brings educational and technological resources and the vaccine directly to the priority communities. CHW interventions provide an evidence-based approach to culturally tailoring messages and addressing social determinant needs that may create barriers to vaccination. With the MHU, CHWs will also be able to rapidly deploy strategies identified through the VCN network for evaluation.

In addition to CHD's promotion of these events, the MHU works with organizations, including consulate offices in different counties, non-profits, and community organizations, to advertise with tailored bilingual messaging that includes the dates/times that the MHU will be in specific areas and locations. The MHU provides services to un/underinsured, farmworker, Latinx, and rural communities throughout Southern Arizona. The MHU does not charge for services and no appointments are necessary. In one Arizona county, arrangements were made with farmworker employers to allow employees to take time off to get vaccinated. The MHU travels across the four Arizona counties along the US-Mexico border. It conducts events during early morning, evening, and weekend hours to increase access. Over the past 5 months, the MHU has averaged 13 events per month.

### Survey development and implementation

The AzVCN created a survey to conduct among patients of the MHU, either at COVID-19 vaccination events or other health events provided by the MHU. The survey contained CDC recommended survey items on COVID-19 vaccine confidence and uptake. The CDC requested all PRCs use their recommended core survey items to assess vaccine confidence and uptake in their priority communities, if feasible (Table 2). The main benefit of using these standardized items is to allow PRCs to compare their findings to CDC estimates for their state and the nation. Additionally, it will help the CDC understand the effectiveness of community engagement strategies used by PRCs to increase vaccine confidence and uptake in various populations. In addition, the AzVCN included optional survey items to assess perceptions of new variants and need for booster vaccinations The AzVCN survey was in process during the end of 2021 and continuing through summer 2022. As of May 1, 2022, 192 surveys of MHU patients had been completed.

**Table 2:** CDC core survey items.

**Construct**	**Question**	**Response scale**	**Source**
BeSD domain: thinking and feeling domain			
Perceived susceptibility	How concerned are you about getting COVID-19?	Not at all concerned A little concerned Somewhat concerned Very concerned	NIS-ACM
Confidence in vaccine effectiveness	How important do you think getting a COVID-19 vaccine is to protect yourself against COVID-19?	Not at all important A little important Somewhat important Very important	NIS-ACM
Confidence in vaccine safety	How safe do you think a COVID-19 vaccine is for you?	Not at all safe Somewhat safe Very safe Completely safe	NIS-ACM
BeSD domain: social processes			
Social norms	If you had to guess, about how many of your family and friends have received a COVID-19 vaccine?	None Some Many Almost all	NIS-ACM
Provider recommendation	Has a doctor, nurse, or other health professional ever recommended that you get a COVID-19 vaccine?	Yes No Not sure	NIS-ACM
Exposure to misinformation	In the last month, have you seen or heard any negative information about the safety or effectiveness of COVID-19 vaccines?	Yes No Not sure	Other
BeSD domain: practical issues			
Perceived access	How difficult [would it be for you / was it for you] to get a COVID-19 vaccine?	Not at all difficult A little difficult Somewhat difficult Very difficult	NIS-ACM
Incentives	Have you heard of cash prizes or other rewards being offered in your area to people who get a COVID-19 vaccine?	Yes No Not sure	Omnibus
Requirements	Does your work or school require you to get a COVID-19 vaccine?	Yes No Unemployed/Not applicable (Not in school, home schooled) Not sure	NIS-ACM
BeSD domain: COVID-19 vaccination			
Behavior	Have you received at least one dose of a COVID-19 vaccine?	Yes No Not sure	NIS-ACM
Behavior	How many doses of a COVID-19 vaccine have you received?	One Two More than two Not sure	NIS-ACM
Brand	Which brand of COVID-19 vaccine did you receive?	Pfizer-Biontech Moderna Johnson & Johnson/ Janssen Other Not sure	NIS-ACM
Intentions	Ask if no vaccine doses received How likely are you to get a COVID-19 vaccine?	Definitely get a vaccine Probably get a vaccine Not sure Probably not get a vaccine Definitely not get a vaccine	NIS-ACM

### Video testimonials

The AzVCN partnered with a non-profit organization in Arizona to create COVID-19 vaccination testimonial videos from respected community members. The RICE is a community-based, non-profit organization dedicated to serving and assisting Refugees, Asylees, and Immigrants in the Phoenix Metropolitan Area, created to close the service gaps that remain after the 90-day resettlement period testimonials in different languages by vaccine ambassadors/champions. In meetings with the AzVCN and RICE leadership, content of the testimonial videos was discussed. Testimonial videos that encourage specific immigrant communities in their native language to get the COVID-19 vaccine was the overarching message. The testimonials from respected community members discussed how they had received the OCVID-19 vaccine, how it affected them, and how it felt to do their part in bringing an end to the pandemic. Testimonial videos were filmed by the MEZCOPH Western Region Public Health Training Center in partnership with the RICE and the respected community members were selected by the RICE. Testimonial videos were created in French, Arabic, Persian, and Burmese and disseminated by RICE and the AzVCN to their networks and media channels.

## Discussion

The AzVCN project activities has practical implications for addressing COVID-19 misinformation and vaccine uptake among underserved communities, especially Latinx rural, border, and farmworker populations. With a focus on providing activities that extends beyond addressing a single barrier, the AzVCN connects participants to COVID-19 information and vaccination opportunities. The CHWs at MHU events have a crucial role to play in COVID-19 vaccination uptake, particularly in addressing structural barriers, informational barriers, and behavioral barriers. The unique situation that US-Mexico border populations face underscores the importance of these project activities. The MHU is an important vehicle to gain access to an invisible population (e.g., the combination of the clinic being through the university and not the government and the partnerships with consulate offices). The non-governmental nature of the MHU with its trusted CHW bilingual and bicultural staff is of great importance for reaching undocumented/mixed status families and the consulate connection allows for binational collaborations to serve a transnational population.

COVID-19 vaccine acceptance among Latinx populations continues to be of high priority. It is encouraging that Latinx groups feel that COVID-19 vaccine endorsements from same-race medical professionals would increase their willingness to receive it and that they would also be motivated by receiving more information on the experiences of COVID-19 vaccine recipients who are of their own race and ethnicity (16). Our experiences with the MHU staff being public health professionals but also trusted community members of the same race and ethnicity addresses these issues related to the health system. In terms of technical issues of the health system, the MHU's ability to be nimble and participate in planned and pop-up events supplements the structural barriers inherent in a non-centralized health system.

This manuscript describes the AzVCN activities in 2021 and 2022 that were designed to facilitate the identification and translation of effective strategies to implement COVID-19 immunization programing. The COVID-19 pandemic is a once in a century event that revealed widening health disparities and disproportionate adverse health outcomes among underserved populations. In Arizona, these included Latinx, farmworker, border, rural, American Indian, and elderly populations. Throughout the pandemic, underserved populations were among the very populations with lower rates of COVID-19 vaccination, especially in the early weeks of Arizona's COVID-19 vaccination rollout. The listening session dialogues led to increased understanding of barriers and facilitators for COVID-19 vaccination and improved collaborations by delineating actions and results with a goal of validating facilitators/barriers in an iterative process. The partnership with the MHU allowed reach into underserved populations and addressed difficult barriers to overcome for these populations, including structural, informational, and behavioral barriers.

### Limitations

Our experience in developing and implementing the activities of this project has some limitations. First, listening sessions were guided by a set of questions and prompts that were developed internally and may not be generalizable to other listening sessions from other PRCs. However, we did attempt to follow a standard set of questions and prompts for each session to be internally consistent and attempt to allow all listening sessions discuss similar items. Second, our events attended for our surveys were subject to student availability to conduct the survey. We attempted to reach as many different MHU events as possible, but there may be some underreporting among particular events.

## Conclusion

As a participatory evaluation project, the AzVCN efforts contributed to the CDC's Vaccinate with Confidence Strategy by collaborating with CHWs and other key stakeholders to engage directly with communities in identifying and addressing barriers to vaccine uptake. By leveraging the MHU to address Latinx COVID-19 vaccination structural barriers and misinformation, especially among undocumented or mixed status families, the AzVCN has made an impact in the COVID-19 vaccine efforts in Arizona. Rural populations in Arizona show increased uptake of COVID-19 vaccinations (17). Further efforts can be informed by actionable plans because of our project, which can include key informant and stakeholder feedback and partnerships with MHUs to address structural and misinformation barriers that will likely continue to exist. By providing a detailed account of our methodology and activities, we show that underserved populations can be reached, and COVID-19 vaccination knowledge and uptake can be impacted positively.

## Data availability statement

The raw data supporting the conclusions of this article will be made available by the authors, without undue reservation.

## Ethics statement

The studies involving human participants were reviewed and approved by University of Arizona Human Subjects Protection Program. Written informed consent for participation was not required for this study in accordance with the national legislation and the institutional requirements.

## Author contributions

TN: conceptualization of project and manuscript, project PI, methodology, formal analysis, roles, writing–original draft, and writing–review and editing. LAS: roles, writing–original draft, conceptualization, and writing–review and editing. AW-L: conceptualization and writing–review and editing. SC: overall PRC PI, roles, writing–original draft, supervision, and validation. JZ, KC, Ras, Res, and MM: roles, writing–original draft, and writing–review and editing. CF: database creation, roles, writing–original draft, and writing–review and editing. MI: project administration, conceptualization and writing–original draft, and writing–review and editing. All authors contributed to the article and approved the submitted version.

## Funding

This manuscript was funded, by the Centers for Disease Control and Prevention (CDC) Award#: 6U48DP006413-02-01.

## Conflict of interest

The authors declare that the research was conducted in the absence of any commercial or financial relationships that could be construed as a potential conflict of interest.

## Author disclaimer

The content is solely the responsibility of the authors and does not necessarily reflect the official views of the CDC.

## Publisher's note

All claims expressed in this article are solely those of the authors and do not necessarily represent those of their affiliated organizations, or those of the publisher, the editors and the reviewers. Any product that may be evaluated in this article, or claim that may be made by its manufacturer, is not guaranteed or endorsed by the publisher.
