# Erratum: The Arizona Prevention Research Center partnerships in Arizona to promote COVID-19 vaccine health equity

**DOI:** 10.3389/fpubh.2022.1031760

**Published:** 2022-09-14

**Authors:** Frontiers Production Office

**Affiliations:** Frontiers Media SA, Lausanne, Switzerland

**Keywords:** COVID-19, vaccine, health equity, Latinx, partnerships, collaborations

Due to a production error, there was an error in the affiliation of Maria Gudelia Rangel Gomez. Instead of “College of the Northern Border, Saudi Arabia,” their affiliation should be “El Colegio de la Frontera Norte, Mexico.”

The publisher apologizes for this mistake. The original version of this article has been updated.

